# Research progress of biomarkers in the prediction of anti-PD-1/PD-L1 immunotherapeutic efficiency in lung cancer

**DOI:** 10.3389/fimmu.2023.1227797

**Published:** 2023-07-03

**Authors:** Luyao Wang, Zongxing Yang, Fucheng Guo, Yurong Chen, Jiarui Wei, Xiangpeng Dai, Xiaoling Zhang

**Affiliations:** ^1^ Key Laboratory of Organ Regeneration and Transplantation of Ministry of Education, First Hospital of Jilin University, Changchun, China; ^2^ National-Local Joint Engineering Laboratory of Animal Models for Human Disease, First Hospital of Jilin University, Changchun, China; ^3^ Department of Clinical Laboratory, First Hospital of Jilin University, Changchun, China

**Keywords:** lung cancer, biomarker, anti-PD-1/PD-L1 immunotherapy, immune checkpoint, dMMR/MSI, CtDNA, bTMB, cytokines among these immune checkpoints

## Abstract

Currently, anti-PD-1/PD-L1 immunotherapy using immune checkpoint inhibitors is widely used in the treatment of multiple cancer types including lung cancer, which is a leading cause of cancer death in the world. However, only a limited proportion of lung cancer patients will benefit from anti-PD-1/PD-L1 therapy. Therefore, it is of importance to predict the response to immunotherapy for the precision treatment of patients. Although the expression of PD-L1 and tumor mutation burden (TMB) are commonly used to predict the clinical response of anti-PD-1/PD-L1 therapy, other factors such as tumor-specific genes, dMMR/MSI, and gut microbiome are also promising predictors for immunotherapy in lung cancer. Furthermore, invasive peripheral blood biomarkers including blood DNA-related biomarkers (e.g., ctDNA and bTMB), blood cell-related biomarkers (e.g., immune cells and TCR), and other blood-related biomarkers (e.g., soluble PD-L1 and cytokines) were utilized to predict the immunotherapeutic response. In this review, the current achievements of anti-PD-1/PD-L1 therapy and the potential biomarkers for the prediction of anti-PD-1/PD-L1 immunotherapy in lung cancer treatment were summarized and discussed.

## 1 Introduction

Lung cancer is the leading cause of cancer death in the world, which is traditionally divided into small cell lung cancer (SCLC) and non-small cell lung cancer (NSCLC) (1, 2). NSCLC accounts for approximately 85% of lung cancer, which mainly includes lung adenocarcinoma (LUAD), lung squamous cell carcinoma (LSCC), and lung large cell carcinoma (LLCC) (2). Surgery, radiation therapy, chemotherapy, targeted therapy, immunotherapy, and combined therapy are current treatment strategies of lung cancer (3). Importantly, the efficiency of these treatment strategies varies from the type and stage of lung cancer. Surgery is the main treatment choice for early-stage (stage I and II) patients, which may provide a longer survival time (4). Primary radiation therapy (such as stereotactic body radiotherapy) is an alternative therapeutic strategy for patients unsuitable for surgery and patients who are medically inoperable (4). However, more than 70% of patients diagnosed with lung cancer are at stage III or IV (5). The standard strategy is adjuvant chemotherapy postoperatively for stage IIIA patients who can benefit from surgery (6). Moreover, the concurrent chemoradiotherapy (CCRT) followed by programmed death 1 ligand (PD-L1) inhibitor treatment is the standard strategy for unresectable stage III lung cancer patients (7).

**Figure 1:**
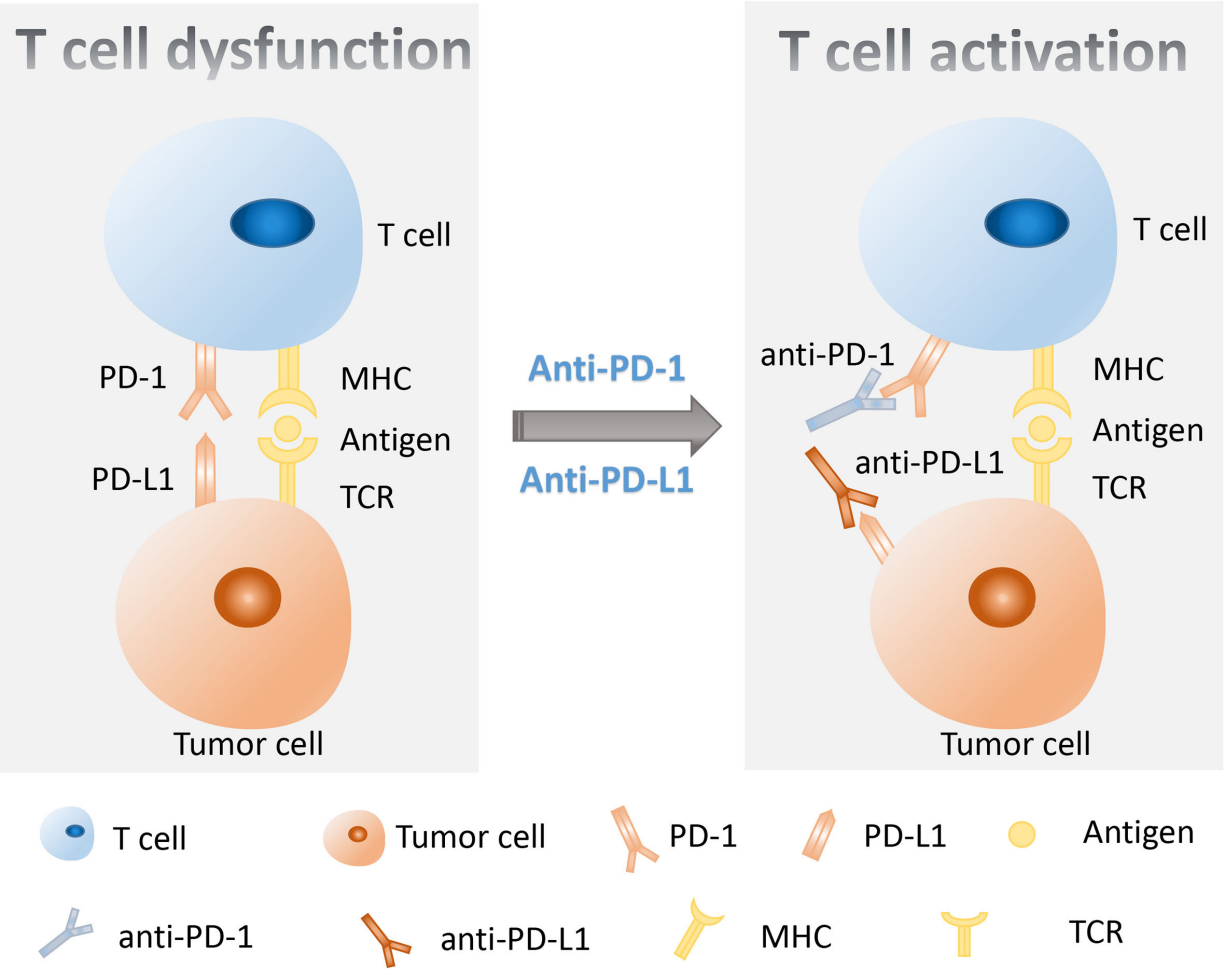
A schematic diagram of the molecular mechanism using anti-PD-1/PD-L1 therapy to restore T-cell functions. PD-1 on T lymphocytes and PD-L1 on tumor cells are two important immune checkpoints that, when combined, transmit inhibitory signals for T-cell activation. The inhibitors of PD-1 or PD-L1 could block the PD-1/PD-L1 axis and rescue the T-cell functions. PD-1, programmed death 1; PD-L1, programmed death 1 ligand.

**Figure 2:**
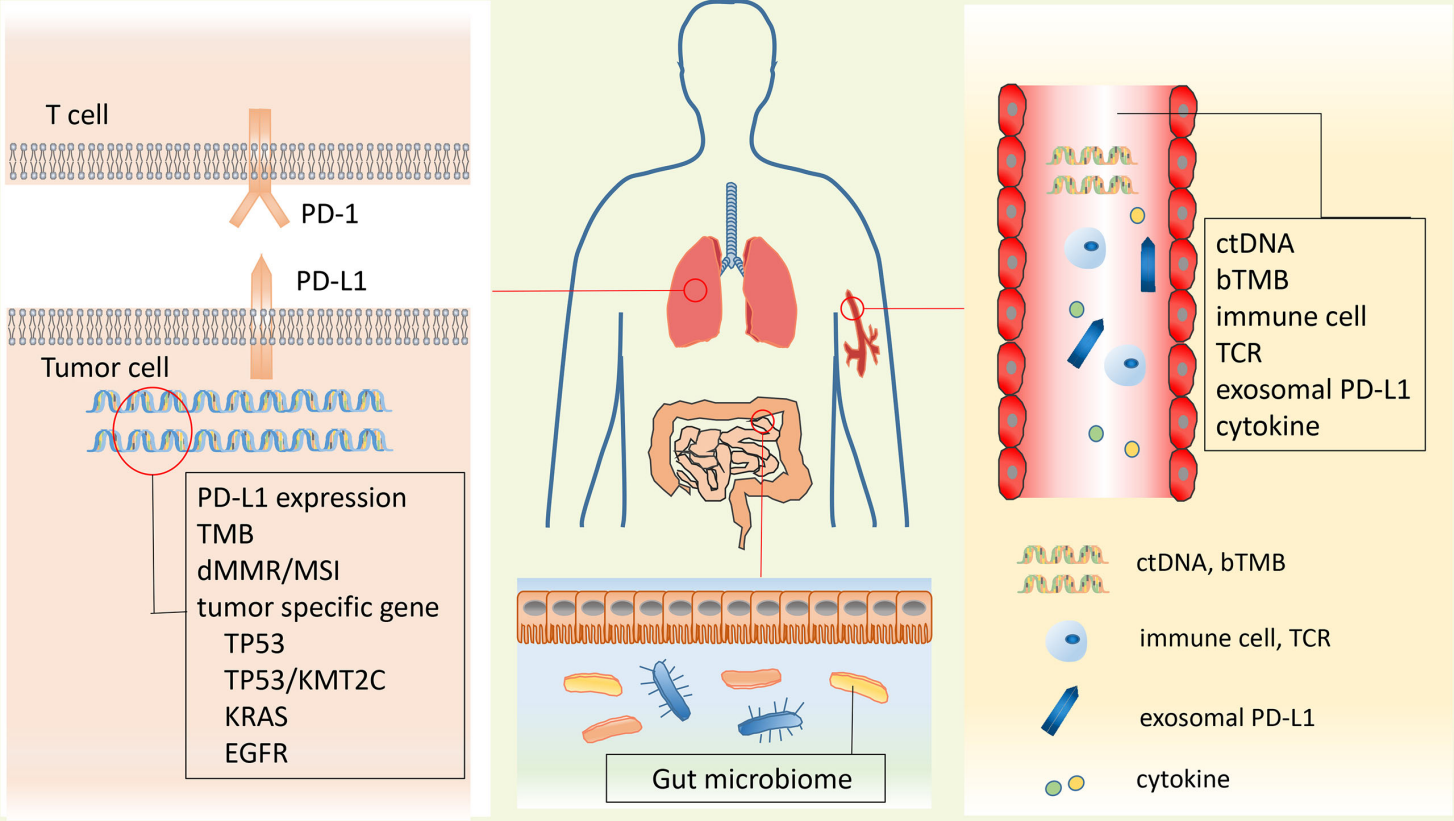
Potential predictive biomarkers of response for anti-PD-1/PD-L1 therapy. Biomarkers could be found from two traditional biopsy samples and the peripheral blood. Biomarkers isolated from traditional biopsy mainly include PD-L1 expression, TMB, dMMR/MSI, and tumor-specific genes (TP53, TP53/KMT2C, KRAS, and EGFR). Peripheral blood biomarkers include ctDNA, bTMB, immune cells, TCR, soluble PD-L1, and cytokines. Gut microbiome is also a promising predictor for immunotherapy in lung cancer. PD-L1, programmed death 1 ligand; TMB, tumor mutation burden; dMMR, MMR deficiency; ctDNA, circulating tumor DNA; bTMB, blood tumor mutation burden; TCR, T-cell receptor.

It was well known that the targeted therapy was the first-line treatment for lung cancer with specific targetable oncogenic drivers, while the platinum-based combination chemotherapy was the standard treatment for lung cancer without specific targetable oncogenic drivers (6). However, in recent years, the immunotherapy was introduced to the treatment of stage IV lung cancer patients and improved the therapeutic effect and survival time of patients (8–10). The immune checkpoint blockade (ICB) therapy including anti-programmed death 1 (PD-1) therapy, anti-programmed death 1 ligand (PD-L1), and anti-cytotoxic-T-lymphocyte-associated protein 4 (CTLA-4) therapy has been proven to be beneficial in some lung cancer patients in clinical trials (8–12). The immune checkpoints are important regulators of the immune system that maintain self-tolerance, protect tissue from damage, and prevent autoimmune responses by modulating the duration and intensity of the immune response in normal states (13). However, the immune checkpoints also affect the antitumor immunity because of their role as mediators in tumor immune evasion. PD-1 on T lymphocytes and its principal ligand PD-L1 on tumor cells are two well-known immune checkpoints that deliver inhibitory signals of T-cell proliferation, cytokine production, and cytotoxicity when they are bound (14). Therefore, blockage of PD-1/PD-L1 by their inhibitors could boost the killing effect of immune system on tumor cells (**Figure 1**). It was confirmed that the anti-PD-1/PD-L1 immunotherapy has shown significant antitumor efficiency and good safety in the treatment of lung cancer (12). Nevertheless, some side effects occurred in the process of lung cancer anti-PD-1/PD-L1 therapy, and some patients do not respond well to their treatment (12). It was reported that the expression of PD-L1, tumor mutation burden (TMB), tumor-specific genes, dMMR/MSI, gut microbiome, and the invasive peripheral blood biomarkers including blood DNA-related biomarkers, blood cell-related biomarkers, and other blood-related biomarkers were potential biomarkers to predict clinical response of anti-PD-1/PD-L1 therapy in cancers (15). Therefore, it is of importance to explore some biomarkers to predict the response to immunotherapy, which is helpful for the precision medicine of lung cancer patients (**Figure 2**).

Here, we summarized and discussed the current achievements of anti-PD-1/PD-L1 therapy and the potential biomarkers for the prediction of anti-PD-1/PD-L1 immunotherapy in lung cancer treatment. Abbreviations and their full names were shown in **Table 1**.

**Table 1:** Summarization of abbreviations and their full name.

Abbreviation	Full name
SCLC	Small cell lung cancer
NSCLC	Non-small cell lung cancer
LUAD	Lung adenocarcinoma
LSCC	Lung squamous cell carcinoma
LLCC	Lung large cell carcinoma
CCRT	Concurrent chemoradiotherapy
PD-1	Programmed death 1
PD-L1	Programmed death 1 ligand
CTLA-4	Cytotoxic-T-lymphocyte-associated protein 4
anti-PD-1	Anti-programmed death 1
anti-PD-L1	Anti-programmed death 1 ligand
anti-CTLA-4	Anti-cytotoxic-T-lymphocyte-associated protein 4
FDA	Food and Drug Administration
ORR	Overall response rate
OS	Overall survival
PFS	Progression-free survival
EGFR	Epidermal growth factor receptor
ALK	Anaplastic lymphoma kinase
TMB	Tumor mutation burden
mut/Mb	Mutations/megabase
IV	Intravenously
TMB-H	TMB-high
ES-SCLC	Extensive-stage SCLC
ICIs	Immune checkpoint inhibitors
WES	Whole exome sequencing
MMR	Mismatch repair
MSI	Microsatellite instability
dMMR	MMR deficiency
VAF	Variant allele fraction
bTMB	Blood TMB
ctDNA	Circulating tumor DNA
TILs	Tumor infiltrating lymphocytes
QIF	Quantitative immunofluorescence
SCFAs	Short chain fatty acids
TME	Tumor microenvironment
CAR-T	Chimeric antigen receptor T
NSqNSCLC	nonsquamous non-small-cell lung cancer
nab	nano-particle albumin-bound
ECOG	Eastern Cooperative Oncology Group
TPS	Tumor Proportion Score

## 2 Mechanisms of anti-PD-1/PD-L1 immunotherapy

Cancer immunotherapy, which functions by leveraging the cytotoxic potential of human immune system to kill cancer cells, has become a powerful strategy for cancer treatment. A large number of antibodies and small molecules targeting immune checkpoints are currently undergoing pre-clinical and clinical studies (16). The immune checkpoint proteins under study mainly include PD-1, PD-L1, CTLA-4, lymphocyte-activation gene 3 (LAG3), and T-cell immunoglobulin and mucin domain 3 (TIM3) (17). Notably, PD-1 and PD-L1 are the focus of all these immune checkpoints, which are also well studied (16). PD-1 is a type I transmembrane protein that is mainly expressed on activated T cells, B cells, and natural killer (NK) cells (18). PD-L1 is a member of the B7 protein family and is mainly expressed on the tumor cells, tumor-infiltrating cells, and antigen-presenting cells (APCs) (19).

The activation of T cells relies on at least two signals. The first signal is the T-cell receptor (TCR) recognition of the antigen presented by the major histocompatibility complex (MHC) antigen in the form of peptides. The second signal is the co-stimulatory signal provided by antigen-presenting cells (APCs), which is generated by the interaction between co-stimulatory ligands on APCs and corresponding receptors on the surface of T cells (20). Co-stimulatory signals are necessary for the induction of productive immune responses as it is essential for the optimal proliferation, differentiation, and survival of T cells. Under physiological conditions, the PD-1/PD-L1 axis is crucial in the development of immune tolerance, which can prevent excessive immune cell activity-induced tissue destruction and autoimmunity by regulating the quantity and activity of antigen-specific T cells (21). However, in tumor environments, the interaction between PD-1 and PD-L1 could inhibit T-cell activation and cause T-cell apoptosis, reduced cytokine production, T-cell lysis, and induction of tolerance to antigen, thus enabling the tumor to evade immune surveillance (22). PD-1 is mainly expressed on activated T cells and can serve as a brake for T-cell activation when combined with its ligands PD-L1. After binding with PD-L1, PD-1 is phosphorylated at its immune receptor tyrosine-based inhibitory motif (ITIM) and immune receptor tyrosine-based switch motif (ITSM), leading to the recruitment of tyrosine phosphatase SHP2 (Src homologous phosphatase 2) and subsequent dephosphorylation of TCR and CD28, thereby inhibiting T cell-related signaling (23–26). In addition to inhibiting some early activation pathways of T cells, a recent study has shown that PD-1 can directly prevent antigen recognition by disrupting the cooperative TCR–pMHC–CD8 trimolecular interaction (27). Under the intervention of PD-1/PD-L1 immune checkpoint inhibitors (ICIs), the membrane motif of PD-1 cannot be phosphorylated, resulting in the inability of cells to recruit SHP-2. Then, the dephosphorylation of TCR and CD28 is blocked, leading to effective transmission of activation signals to downstream proteins and signaling pathways, ultimately stimulating T-cell proliferation and differentiation. Ultimately, the immune function of T cells is effectively exerted.

## 3 Mechanisms of resistance for anti-PD-1/PD-L1 immunotherapy

In recent years, anti-PD-1/PD-L1 immunotherapy has achieved surprising effects in the treatment of various malignant tumors (28, 29). However, many patients have developed resistance to PD-1/PD-L1 inhibitors, which severely limits the wide application of this therapy and has become a serious clinical problem in this field. Therefore, it is necessary to deeply reveal the molecular mechanism of PD-1/PD-L1 inhibitor resistance and therefore improve the response rate of cancer patients to anti-PD-1/PD-L1 immunotherapy.

The resistance to anti-PD-1/PD-L1 therapy can be classified into primary resistance and acquired resistance based on clinical outcomes (30). In primary resistance, patients failed to exhibit clinical response when treated with PD-1/PD-L1 inhibitors (30). In contrast, acquired resistance means that patients respond to anti-PD-1/PD-L1 therapy at the beginning of treatment, but then the therapeutic effect of the therapy is significantly weakened or unresponsive (30). The mechanism of primary resistance mainly includes lack of immunogenic antigens (31), restriction of T-cell infiltration (32), lack of interferon responsiveness (33), abnormal gut microbiome composition (34), epidermal growth factor receptor (EGFR) mutations, or anaplastic lymphoma kinase (ALK) rearrangements (35). The mechanism of acquired resistance may be associated with the weakened recognition of tumor antigens by immune cells, the loss of neoantigen, the change of the tumor microenvironment (TME), and aberrant cellular signaling transduction (18, 19).

Tumor cells can avoid processing and presenting tumor antigens by silencing or altering the expression of antigen-presenting machinery, beta-2-microglobulin (B2M), or major histocompatibility complex (MHC) molecules (36). B2M plays an important role in supporting MHC class I molecules to present tumor-specific peptide antigens to T cells. The loss of functional B2M may be a mechanism of tumor resistance to T cell-mediated immune responses (37).

Neoantigen loss is also a mechanism of acquired resistance to immune checkpoint therapy (38). The analyses on matched pretreatment and resistant tumors showed that resistant clones lost 7 to 18 putative mutation-associated neoantigens (38). This result proved that the loss of neoantigen could augment acquired resistance as an escape mechanism after PD-1/PD-L1 blockade therapy (38).

In immunosuppressive TME, tumor cells can interact with the stromal cells and immune cells to prevent immune surveillance and immune system killing (39). In TME, in addition to tumor cells, other components that might be associated with primary or acquired resistance include exhausted T cells, T regulatory cells, myeloid-derived suppressor cells (MDSCs), macrophages, immunosuppressive cytokines, and gut microbiome (40). Moreover, the alterations of metabolic landscape of the TME could also suppress the infiltration of immune cells and other antitumor immune functions by producing immunosuppressive metabolites (41). For primary resistance, a study has shown that TGFβ shaped the TME to attenuate tumor response by restricting T-cell infiltration in anti-PD-L1 therapy (32).

Abnormal cellular signaling transduction is also an important factor leading to immunotherapy resistance. For example, the IFN-γ pathway has been proved to be associated with resistance to checkpoint blockade therapy (42). Interferon-γ produced by tumor-specific T cells that have recognized their cognate antigen on tumor cells or APCs could induce effective antitumor immune response. Lack of IFN responsiveness resulted in resistance to anti-PD-1 therapy (33). However, the duration of tumor interferon signaling allowed immuno-editing of tumors and resulted in adaptive resistance to immune checkpoint blockade therapy (42).

The abnormal gut microbiome composition may be responsible for primary resistance to immune checkpoint blockade therapy. A study has shown that antibiotics reduced the clinical benefit of immune checkpoint blockade therapy for patients with advanced cancer (34). The antitumor effects of PD-1 blockade therapy on germ-free or antibiotic-treated mice can be enhanced by performing fecal microbiota transplantation (FMT) from cancer patients who responded to ICIs, but not from the nonresponding patients (34). Metagenomics of patient stool samples at diagnosis showed that the clinical response of patients to ICIs was related to the relative abundance of Akkermansia muciniphila (34).

EGFR mutations and ALK rearrangements are associated with primary resistance to PD-1/PD-L1 blockade therapy in NSCLC. A retrospective analysis evaluated the efficacy of anti-PD-1/PD-L1 therapy on EGFR-mutant, ALK-positive, and EGFR wild-type/ALK-negative patients who received anti-PD-1/PD-L1 therapy (35). The results revealed that the NSCLC patients who harbored EGFR mutations or ALK rearrangements are associated with low objective response rates to anti-PD-1/PD-L1 therapy, which may be due to low rates of co-localized PD-L1 expression and CD8(+) tumor-infiltrating lymphocytes (TILs) (35).

## 4 Anti-PD-1 immunotherapies for lung cancer

### 4.1 Nivolumab

Nivolumab, a fully human antibody targeting PD-1, has been approved for the treatment of several cancers including lung cancer by the Food and Drug Administration (FDA) (43). In 2015, nivolumab was approved as the second-line treatment strategy for advanced squamous NSCLC patients with progression during or after platinum-based chemotherapy based on the CheckMate 017 study (44). The median overall survival (OS) of patients treated with nivolumab was 9.2 months, which is longer than that of the patients treated with docetaxel (6.0 months), proving the positive effect of nivolumab for patients with advanced squamous NSCLC. In 2020, the combination therapy of nivolumab and ipilimumab (an CTLA-4 inhibitor) was approved for the treatment of patients with metastatic NSCLC without epidermal growth factor receptor (EGFR) or anaplastic lymphoma kinase (ALK) genomic aberrations (45, 46). The CheckMate-9LA study is a randomized and open phase III study that involved stage IV or recurrent NSCLC patients (45, 46). This study compared the clinical benefit of combination therapy (nivolumab plus ipilimumab with two cycles of chemotherapy) and chemotherapy alone. Compared with 10.7 months median OS of chemotherapy, the combination of nivolumab plus ipilimumab with two cycles of chemotherapy improved OS to 11.4 months. The approval of the CheckMate-9LA regimen benefited from the safety and efficacy data of CheckMate 568 and CheckMate 227 (47, 48). Moreover, CheckMate 568 is an open phase II study and aimed to evaluate the efficacy and safety of nivolumab combined with ipilimumab in the treatment of advanced/metastatic NSCLC, and to investigate the correlation between PD-L1 expression and TMB on the treatment efficacy (47). The results proved the safety and effectiveness of the combination and also indicated that 10 or more mutations/megabase (mut/Mb) of TMB were associated with better response and longer progression-free survival (PFS) regardless of PD-L1 expression (47). In line with this finding, the CheckMate-227 study was performed as an open phase III study. The results indicated that the combination of nivolumab and ipilimumab exhibited a longer OS time than chemotherapy in NSCLC patients without causing new safety concerns (48).

In 2022, FDA approved the combination of nivolumab and chemotherapy as adjuvant therapy for resectable NSCLC based on CheckMate 816 clinical trials (49). In the study, stage IB to IIIA resectable NSCLC patients were treated with nivolumab and platinum-based chemotherapy or platinum-based chemotherapy alone before resection. The median event-free survival of the combination of nivolumab and platinum-based chemotherapy was 31.6 months, compared with the 20.8 months of chemotherapy alone. Importantly, compared with the 2.2% pathological complete response for chemotherapy alone, the pathological complete response was 24.0% for the patients who received the combined therapy. Moreover, the combined therapy did not increase adverse events or hinder the surgery feasibility. Therefore, the combination of nivolumab and platinum dramatically improved the clinical benefit of treatment for patients with resectable NSCLC compared with chemotherapy alone.

Furthermore, nivolumab was also approved for the treatment of SCLC as a salvage regimen (43). In the CheckMate-032 study, the efficiency and safety of nivolumab as a monotherapy or in combination with ipilimumab (an CTLA-4 inhibitor) in the treatment of multiple types of tumors were evaluated (43). Those SCLC patients who failed in previous platinum-based chemotherapy were treated with nivolumab or a combination of nivolumab and ipilimumab. The overall response rate (ORR) for the single treatment of nivolumab and the combination of nivolumab and ipilimumab was 10% and 23%, respectively. However, the median OS was 4.4 months and 7.7 months, respectively. These results indicated that both the monotherapy of nivolumab and the combination therapy of nivolumab and ipilimumab showed significant efficacy in the treatment of SCLC.

### 4.2 Pembrolizumab

Pembrolizumab, the humanized monoclonal antibody targeting and blocking the PD-1, was approved as the second-line treatment for advanced NSCLC in 2015 based on the KEYNOTE-001 study, which aimed to assess the efficacy and safety of pembrolizumab in the treatment of NSCLC (50–52). The inclusion criteria of this study are the locally advanced or metastatic non-small cell lung cancer patients with or without treatment previously. The study presented 22.3 months and 10.5 months median OS for the naive patients and previously treated patients, respectively. Therefore, pembrolizumab provided acceptable antitumor activity and tolerable safety for the treatment of patients with advanced NSCLC, paving the way for the FDA approval (52, 53). Importantly, in 2016, pembrolizumab was approved as the first-line treatment of NSCLC patients with high PD-L1 expression based on the KEYNOTE-024 trial, which aimed to test its therapeutic effect on metastatic treatment-naive NSCLC (51, 54, 55). In this clinical trial, 154 previously treatment-naive patients with advanced NSCLC received pembrolizumab treatment and at least 50% of tumors cells of these patients expressed PD-L1 without EGFR or ALK genomic aberrations. The other 151 patients received platinum-based chemotherapy. Conclusively, the PFS of the pembrolizumab group and chemotherapy group was 10.3 months and 6.0 months, respectively. Furthermore, the pembrolizumab group exhibited a better OS and improved response rate. Moreover, the serious adverse events were 56.6% and 26.6% in the chemotherapy group and in the pembrolizumab group, respectively (55).

In 2017, FDA approved the combination of pembrolizumab, pemetrexed, and carboplatin for the treatment of patients with previously untreated metastatic NSCLC based on the KEYNOTE-021 study (56). The results of this study indicated that the combination group (pembrolizumab plus chemotherapy) achieved better ORR and PFS than the chemotherapy group. Moreover, in 2018, based on the results of the KEYNOTE-189 study, the combination of pembrolizumab, pemetrexed, and platinum was approved as the first-line treatment for the patients with metastatic non-squamous non-small-cell lung cancer (NSqNSCLC) and the patients lack EGFR or ALK genomic tumor aberrations (57). The results from the KEYNOTE-189 study showed that the combination of pembrolizumab, pemetrexed, and platinum dramatically increased the ORR and PFS. In 2018, based on the clinical results of the KEYNOTE-407 study, FDA also approved the combination of pembrolizumab, carboplatin, and paclitaxel or nab-paclitaxel as first-line treatment for metastatic squamous NSCLC (58). The results showed that the median OS of the pembrolizumab combination group was 15.9 months, which is longer than that of the placebo combination group (11.3 months). Moreover, the median PFS was 6.4 months in the pembrolizumab combination group and 4.8 months in the placebo combination group. Therefore, these results indicated that the application of pembrolizumab in chemotherapy with carboplatin and paclitaxel or nab-paclitaxel significantly improved clinical benefit. In 2019, based on the study of KEYNOTE-024, FDA approved pembrolizumab as first-line treatment for patients with stage III NSCLC (59). The results showed that compared with platinum-based chemotherapy, pembrolizumab treatment significantly prolonged the OS of patients, suggesting that pembrolizumab treatment was a reasonable treatment option for NSCLC patients with low PD-L1 TPS and without EGFR mutation or ALK translocation.

Interestingly, in 2019, pembrolizumab was approved for the treatment of patients with metastatic SCLC based on clinical results of the KEYNOTE-028 trial and the KEYNOTE-158 trial (60). The KEYNOTE-028 trial was a phase Ib trial that aimed to study the tolerability and efficiency of pembrolizumab on 20 tumor types including SCLC (61). In this study, they found a 33.3% ORR, 1.9 months of median PFS, and 9.7 months of median OS. In the phase II KEYNOTE-158 study, SCLC patients were treated by 200 mg of pembrolizumab every 3 weeks, and the ORR was 18.7% and median PFS was 2 months (61). Moreover, Hyun et al. performed a pooled analysis of KEYNOTE-028 and KEYNOTE-158 trials and found that pembrolizumab exhibited a durable antitumor activity in patients with recurrent or metastatic SCLC who had received two or more previous lines of therapy with good tolerance (62). Furthermore, pembrolizumab was approved for the treatment of patients with unresectable or metastatic TMB-high (TMB-H) solid tumors (≥10 mut/Mb) and yielded a 29% ORR of the TMB-H SCLC patients (63).

### 4.3 Cemiplimab

Cemiplimab, a human PD-1 monoclonal antibody that binds to PD-1 and blocks its interaction with PD-L1 and PD-L2, has been approved as monotherapy for advanced NSCLC with PD-L1 expression in more than 50% tumor cells (64, 65). The phase III study EMPOWER-Lung 1 provided clinical data for the treatment of cemiplimab in advanced NSCLC with PD-L1 expression in at least 50% tumor cells (66). In this study, patients were treated with cemiplimab or platinum-doublet chemotherapy and the transition from chemotherapy to cimilizumab was allowed when disease progressed. The median PFS of the cemiplimab group and chemotherapy group was 8.2 months and 5.7 months, respectively. Compared with chemotherapy, cemiplimab monotherapy significantly improved the OS and PFS of NSCLC patients (at least 50%).

## 5 Anti-PD-L1 immunotherapies for lung cancer

### 5.1 Atezolizumab

Atezolizumab, a fully humanized monoclonal antibody targeting PD-L1, is the first FDA-approved PD-L1 inhibitor for the second-line therapy of NSCLC patients (51). The clinical study NCT01375842 was performed to evaluate the tolerability, safety, and pharmacokinetics of atezolizumab in the treatment of several cancers (67). In this study, NSCLC patients treated with atezolizumab showed 28% of 3-year survival rates, which proved that atezolizumab was well tolerated and had long-term clinical benefits in the treatment of NSCLC patients.

In 2018, FDA approved atezolizumab in combination with bevacizumab, paclitaxel, and carboplatin as the first-line treatment of metastatic non-squamous NSCLC with wild-type EGFR and ALK (68). Results of the IMPOWER150, a randomized, open-label, phase III study, indicated that patients treated with the combination of the four-drug regimen had improved survival rate compared with patients treated with the combination of bevacizumab, carboplatin, and paclitaxel (68). Furthermore, atezolizumab was approved in combination with paclitaxel and carboplatin to treat metastatic non-squamous NSCLC without EGFR or ALK genomic aberrations based on the IMpower-130, which was a multicenter, randomized, open-label, phase III trial and aimed to assess the clinical efficacy and safety of atezolizumab in combination with chemotherapy versus chemotherapy alone to treat non-squamous NSCLC (69). The median OS of patients treated by atezolizumab in combination with chemotherapy was 18.6 months, and it was 13.9 months for the patients treated by chemotherapy alone. Moreover, the median PFS of the combination group was 7.0 months, and it was 5.5 months for the chemotherapy treatment group. Together, the significant improvement of median OS and median PFS in atezolizumab plus chemotherapy group led to the FDA approval of atezolizumab.

Importantly, the atezolizumab is also the first ICI that was approved to treat extensive-stage SCLC (ES-SCLC) in combination with carboplatin and etoposide (70). In the multinational IMpower133 trial, 403 previous untreated patients with ES-SCLC were divided into two groups, one is the atezolizumab group in which the patients received the combination treatment of carboplatin, etoposide, and atezolizumab, and the other is the placebo group in which the patients received the combination treatment of carboplatin, etoposide, and placebo (71, 72). At a median follow-up of 13.9 months, the median OS of the atezolizumab group was 12.3 months, and it was 10.3 months for the placebo group. Accordingly, the median PFS of the atezolizumab group and placebo group was 5.2 months and 4.3 months, respectively. Therefore, the combination of atezolizumab and chemotherapy resulted in improved clinical benefit compared with chemotherapy alone as the first-line treatment of ES-SCLC.

### 5.2 Durvalumab

Durvalumab is a human IgG1 monoclonal antibody that can block the PD-L1 to restore T-cell activity (73). In 2018, durvalumab was applied in the treatment of patients with unresectable stage III NSCLC without progression after platinum-based concurrent chemoradiotherapy (cCRT) (74, 75). The data of the PACIFIC study indicated that durvalumab treatment significantly improved PFS of patients; therefore, durvalumab could be applied as a maintenance therapeutic strategy after chemoradiotherapy for patients with advanced unresectable stage III lung cancer (74, 75). Furthermore, in 2020, based on the CASPIAN study, durvalumab was approved in combination with chemotherapy as the first-line therapy to treat patients suffering from extensive stage small cell lung cancer (76). In the study, ES-SCLC patients who had not received first-line chemotherapy were treated by combination therapy or chemotherapy alone. The median OS of patients treated by durvalumab and platinum-based chemotherapy was 13.0 months, while it was 10.3 months for the patients who received chemotherapy alone. Furthermore, they observed 34% and 25% of 18-month survival rates for these two groups, respectively, which proved the advantage of the combination of durvalumab and platinum-based chemotherapy in the treatment of SCLC.

## 6 Potential predictive biomarkers for anti-PD-1/PD-L1 immunotherapy of lung cancer

Although anti-PD-1/PD-L1 therapy has demonstrated clinical benefits in the treatment of lung cancer, only a limited proportion of patients would benefit from the immunotherapy. For example, only 20%–25% of patients with NSCLC showed a sustainable response to ICIs (77). Therefore, it is urgent and important to identify effective predictive biomarkers for patients suffering from cancer before they are given immunotherapy.

### 6.1 PD-L1 expression

It was reported that the level of PD-L1 expression might be a biomarker for the prediction of the patient response to anti-PD-1/PD-L1 immunotherapy in clinical trials (53, 78, 79). Some results suggested that the high expression of PD-L1 is associated with increased response rates and clinical benefits of anti-PD-1/PD-L1 therapy (80, 81). In the Keynote-001 study, pembrolizumab treatment resulted in a longer median OS for the advanced NSCLC patients with a PD-L1 proportion score of ≥50% than those with a proportion score of 1–49% (53). The Keynote-052 study indicated that the subgroup with PD-L1 expression above 10% showed a higher objective response rate than the subgroup with PD-L1 expression below 1% (39% vs. 11%) in urothelial cancer patients treated with pembrolizumab (82). However, not all patients with high PD-L1 expression will respond well to anti-PD-1/PD-L1 immunotherapy while some patients with negative PD-L1 expression can also benefit from this therapy (83, 84), which indicated that there remain challenges in defining the predictive function of PD-L1 expression. The possible reasons might be as follows: (1) the methods and antibodies used for IHC to detect the PD-L1 level vary from different clinical studies; (2) the scoring system determining the quantification of PD-L1 expression of tumor cells, tumor-infiltrating immune cells, or both, is not consistent and it is difficult to confirm the best cutoff value; and (3) the expression of PD-L1 suffers from heterogeneity in space and time. Studies also showed that the expression of PD-L1 differs in primary and metastatic tumor sites and the PD-L1 expression may be affected by previous chemotherapy (85, 86). The accuracy of histological specimen may be affected by the small size of biopsy tissue (87). Despite these disadvantages, PD-L1 expression remains a promising predictive biomarker for anti-PD-1/PD-L1 immunotherapy.

### 6.2 TMB

TMB, the total number of mutations (including synonymous and non-synonymous) in tumor cells, could be predictive biomarkers for immunotherapy. Advanced sequencing technologies can better characterize the mutational landscape to identify patients who are more likely to gain clinical benefits from immunotherapy. Rizvi et al. performed whole exome sequencing (WES) on NSCLC samples from the patients treated with pembrolizumab and found that in the process of pembrolizumab treatment, the patients having tumors with a high asynchronous mutation burden exhibited improved objective response and a lasting clinical benefit (88). Yarchoan et al. also observed a significant correlation between the tumor mutational burden and the objective response rate in anti-PD-1/PD-L1 therapy against multiple cancer types (89). Importantly, the patients with high TMB and high PD-L1 expression (>50%) had the longest PFS and OS compared with patients with only a single factor, suggesting that the integration of TMB with PD-L1 expression might be more precise to identify the patients who are more likely to respond to anti-PD-1/PD-L1 therapy (90). However, the assessment of TMB needs large sequencing panels that require large amounts of tumor tissue (91). The limited amount of DNA obtained from a conventional tumor biopsy or a fine needle biopsy may make TMB evaluation challenging or even impossible (91). Moreover, the standardization of TMB assessment has not been determined. Although the terms “low TMB” or “high TMB” are commonly used in study, the threshold to define them has not been clearly established. Therefore, the limitations that prevent TMB from becoming a promising predictive marker for immunotherapy should be overcome before it can be used in a clinical setting.

### 6.3 dMMR/MSI

DNA mismatch repair (MMR) is a system that aims to identify and repair mutations that occurred during DNA replication and recombination. Dysfunction of the DNA mismatch repair system will lead to the accumulation of mutations. Moreover, the microsatellite instability (MSI) is a genetically hyper-mutational state that is a phenotypic result of MMR deficiency (dMMR). Studies have shown that MSI-H/dMMR can predict the response to ICIs in patients with colon cancer and endometrial cancer (92, 93). Recently, the role of the MMR system in the response to ICIs in NSCLC was assessed and the alteration of MMR system-related genes in NSCLC seems to be related to the enhanced response to nivolumab immunotherapy (94).

### 6.4 Tumor-specific genes

It was reported that the tumor-specific driver gene mutations are associated with the efficiency of immunotherapy (95). Wang et al. fully analyzed the clinical, genomic, and transcriptomic data of lung adenocarcinoma patients in a public database and evaluated the impact of TP53 variant allele fraction (VAF) on tumor immune microenvironment and clinical outcomes of LUAD patients treated with ICIs (96). They found that compared with patients from the high TP53 VAF group and the wild-type group, low TP53 VAF group patients demonstrated more immune cell infiltration and superior response to anti-PD-1/PD-L1 therapy, proving that low TP53 VAF could be a predictive marker for better clinical outcomes of anti-PD-1/PD-L1 therapy (96). Notably, the KMT2C/TP53 co-mutations might be a promising predictive marker of clinical benefit for immunotherapy due to the high correlation of KMT2C/TP53 co-mutations with naive CD8+ T cells, Th1, Th2, and gd T cells (97). In line with this notion, it was found that KRAS mutation was associated with an inflammatory TME and tumor immunogenicity, leading to higher response of patients to anti-PD-1/PD-L1 immunotherapy in NSCLC patients (98). Interestingly, EGFR mutation is reported to be associated with decreased PD-L1 expression, low TMB, and decreased CD8+ T-cell infiltration (99). In a clinical trial, pembrolizumab showed no effect on the treatment of advanced NSCLC patients with EGFR-mutant and positive PD-L1 expression (≥1%) (100). Comprehensive analysis of EGFR mutation as a predictive biomarker of immunotherapy should be widely carried out in NSCLC (101).

### 6.5 Peripheral blood-related biomarkers

#### 6.5.1 ctDNA and bTMB

Blood DNA-related biomarkers mainly include circulating tumor DNA (ctDNA) and blood TMB (bTMB). The amount of circulating tumor DNA in plasma is related to the tumor burden and clinical outcome (102). It was reported that pretreatment ctDNA is associated with the durable clinical benefit of NSCLC patients treated with ICIs (103). Using ctDNA to detect TMB in peripheral blood has also been developed to estimate response to immunotherapy. Gandara et al. found that TMB in peripheral blood (bTMB) can identify patients who had the clinical improvement in progression free survival after atezolizumab treatment, proving that high bTMB may be a marker for immunotherapy efficiency in NSCLC (104).

#### 6.5.2 Immune cells and TCR

Blood cell-related biomarkers mainly include immune cells and T-cell receptor (TCR) immunophenotyping. Notably, the type and number of immune cells and TCR immunophenotyping can reflect the treatment effect of anti-PD-1/PD-L1 immunotherapy (105, 106). Immune cells in peripheral blood can reflect the functions and subtypes of tumor-infiltrating lymphocytes (TILs). The paired whole exome DNA sequencing and multiplexed quantitative immunofluorescence (QIF) were performed in pretreatment samples from NSCLC patients treated with anti-PD-1 immunotherapy to figure out the role of intratumoral T cells and their relationship with the tumor genomic landscape (107). The results elucidated that the level of CD3+ TILs was related to a favorable response of patients to immunotherapy (107). Furthermore, Han et al. performed sequencing of complementarity-determining region 3 of TCRβ chains isolated from PD-1+ CD8+ T cells to evaluate its value as a biomarker to predict the response to anti-PD-1/PD-L1 therapy in NSCLC patients (108). Those results showed that the TCR diversity and clonality of PD-1+ CD8+ T cells in peripheral blood may be promising predictors of response to anti-PD-1/PD-L1 therapy and survival outcomes in NSCLC patients (108).

#### 6.5.3 Exosomal PD-L1 and cytokines

Exosomal PD-L1 and cytokines are other important blood cell-related biomarkers. PD-L1 was present on exosomes isolated from the plasma of NSCLC patients, and the PD-L1-positive exosomes can impair immune functions by inhibiting cytokine secretion and inducing apoptosis of CD8+ T cells in lung cancer patients (109). In support of this notion, Wang et al. analyzed the blood samples of 149 NSCLC patients and found that the exosomal PD-L1 was correlated with the clinical response of patients to ICI treatment, implying that exosomal PD-L1 could be used for the evaluation of immunotherapeutic efficacy in lung cancer (110). Furthermore, a prospective study of 26 NSCLC patients who received pembrolizumab or nivolumab treatment was conducted (111). The values of IFN-γ, TNF-α, IL-1β, IL-2, IL-4, IL-5, IL-6, IL-8, IL-10, and IL-12 were measured by flow cytometry at the time of diagnosis and at 3 months after initiation of anti-PD-1 therapy (111). Their results showed that high levels of cytokines including IFN-γ, TNF-α, IL-1β, IL-2, IL-4, IL-6, and IL-8 are associated with improved response to immunotherapy and prolonged OS of NSCLC patients (111). Moreover, the other study reported that the elevated baseline serum IL-8 levels are associated with poor outcome in NSCLC patients who received ICIs (112). The above results together showed that the cytokine levels may be potential predictive biomarkers in selecting NSCLC patients who can benefit from anti-PD-1/PD-1 immunotherapy.

### 6.6 Gut microbiome

Accumulating lines of evidence indicated that gut microbiota could regulate the host response to chemotherapeutic drugs and is related to the pharmacological effects of chemotherapies and immunotherapies such as anti-PD-L1 and anti-CLTA-4 therapies (113, 114). Takada et al. performed a retrospective study for 294 patients with advanced or recurrent NSCLC who received nivolumab or pembrolizumab monotherapy at three medical centers in Japan (115). Their univariate analyses indicated that the utilization of probiotics was associated with better disease control and overall response of NSCLC patients with anti-PD-1 therapy (115). These results implied that the gut microbiota might be a novel predictor of clinical response to anti-PD-1/PD-L1 immunotherapy in NSCLC. In accordance, a metabolomics analysis was conducted to detect volatile and non-volatile metabolites of the gut microbiota in 11 NSCLC patients who received nivolumab, and the results showed that 2-Pentanone (ketone) and tridecane (alkane) were associated with early progression, while short-chain fatty acids (SCFAs) (i.e., propionate, butyrate), lysine, and nicotinic acid were associated with long-term clinical benefit, suggesting that gut microbiota metabolic pathways may play an important role in clinical response to immunotherapy (116). However, further in-depth studies are warranted to validate the clinical significance of the gut microbiota as a biomarker of immunotherapeutic efficacy.

## 7 Discussion

**Figure 3:**
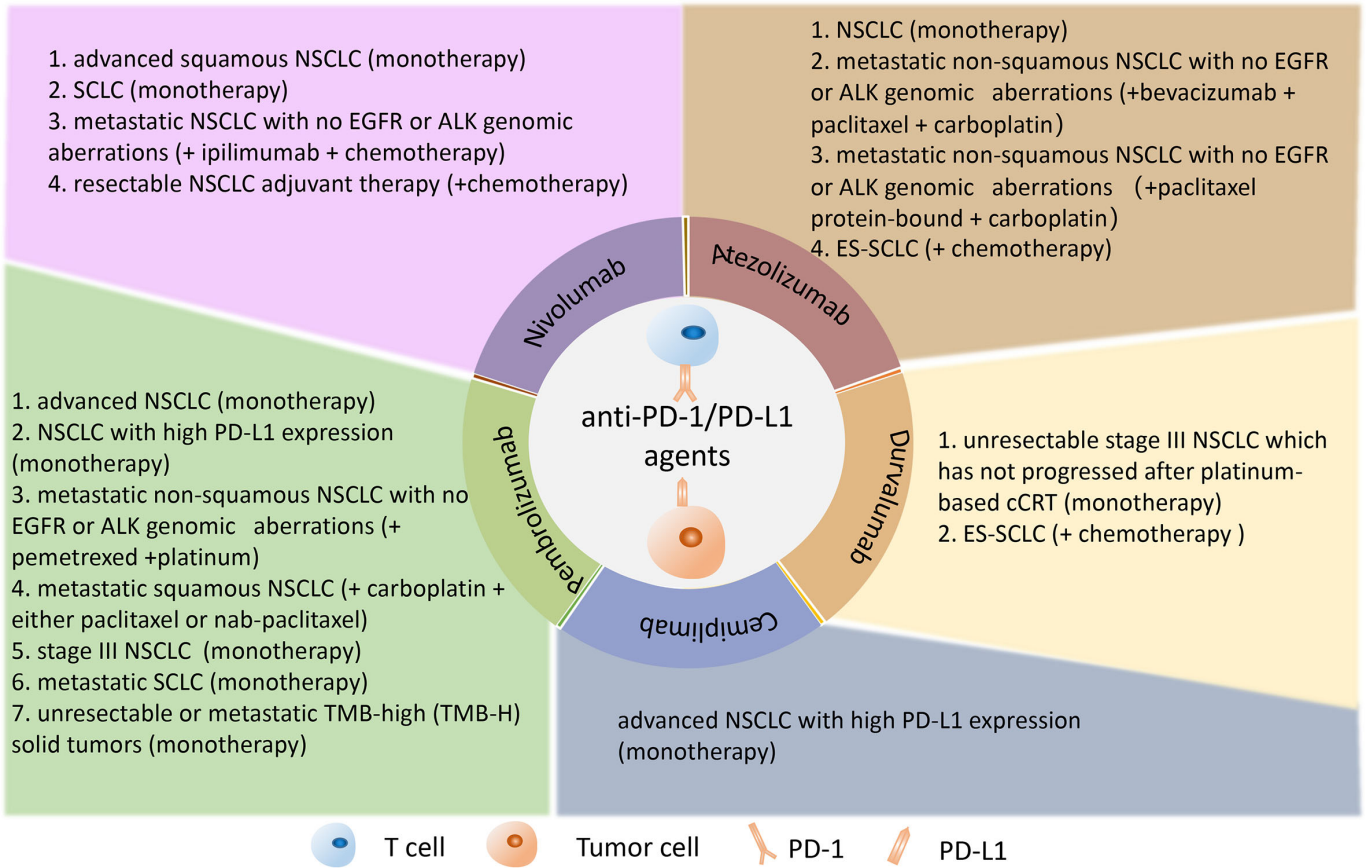
Anti-PD-1/PD-L1 inhibitors are approved by FDA for the treatment of lung cancer. The PD-1 inhibitors such as nivolumab, pembrolizumab, and cemiplimab and the PD-L1 inhibitors such as atezolizumab and durvalumab were illustrated in the panel. PD-1, programmed death 1; PD-L1, programmed death 1 ligand.

Recently, the anti-PD-1/PD-L1 immunotherapy has shown great clinical efficacy in many cancers including lung cancer. Anti-PD-1 drugs, including nivolumab, pembrolizumab, and cemiplimab, and anti-PD-L1 drugs, including atezolizumab and durvalumab, provided significant antitumor activity for lung cancer and improved the survival time of lung cancer patients (**Figure 3**). Although immune checkpoint blockade has achieved clinical success in cancer treatment and several drugs have been approved by FDA to treat cancers, a great proportion of patients could not exhibit sustained clinical response to the anti-PD-1/PD-L1 immunotherapy (117). Current research on anti-PD-1/PD-L1 has shown that some patients who have high PD-L1 expression cannot achieve ideal therapeutic benefit, while some patients who have lower or even negative PD-L1 expression can benefit from anti-PD-1/PD-L1 therapy (18). It is important to explore suitable biomarkers for prediction of immune efficacy and screen suitable patients for anti-PD-1/PD-L1immunotherapy. Here, we summarized and comprehensively discussed the current achievements of anti-PD-1/PD-L1 therapy and the potential biomarkers for the prediction of anti-PD-1/PD-L1 immunotherapy in lung cancer.

**Figure 4:**
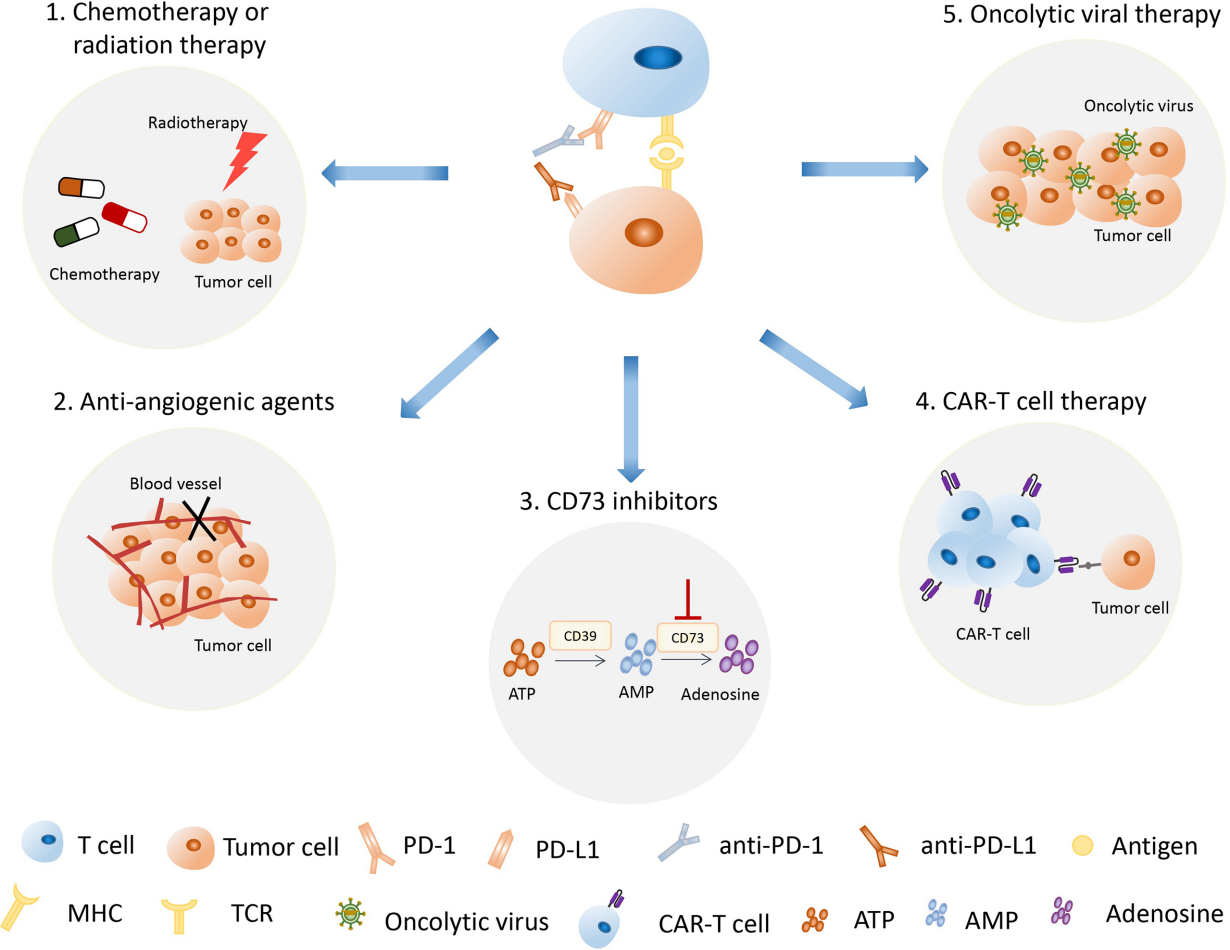
The combination of anti-PD-1/PD-L1 therapy with other therapies has become an important treatment strategy. The strategies used in combination with anti-PD-1/PD-L1 therapy include radiotherapy, chemotherapy, oncolytic viral therapy, anti-angiogenic therapy, CAR-T therapy, and CD73 inhibition therapy. PD-1, programmed death 1; PD-L1, programmed death 1 ligand; CAR-T therapy, chimeric antigen receptor T therapy.

A previous study showed that a large proportion of patients cannot demonstrate sustained clinical response to anti-PD-1/PD-L1 immunotherapy (117). To conquer the problem of low response in patients treated with single immunotherapy, the combination therapy with anti-PD-1/PD-L1 therapy has been developed and used in clinical trials, and they exhibited synergized clinical efficacy and decreased the immune toxicity (**Figure 4**). Notably, radiotherapy (RT) can not only kill tumor cells by damaging the tumor DNA, but also stimulate the immune system by releasing tumor antigens (118). Moreover, preclinical studies indicated that radiotherapy could upregulate PD-L1 expression on tumor cells, which synergistically improved antitumor effect when combined with anti-PD-L1 therapy (119, 120). The combination therapy of RT and anti-PD-1/PD-L1 therapy proved to be a safe strategy by the clinical trial of 187 NSCLC patients (121). In support of this notion, studies have shown that chemotherapy may enhance the efficiency of anti-PD-1/PD-L1 therapy by inducing a tumor-specific adaptive immune response (122). The combined treatment of atezozumab, carboplatin, and etoposide, and the combined treatment of duvarumab and chemotherapy have been successively approved by FDA as the first-line treatment of ES-SCLC (70, 76). Recently, the combination of nivolumab and chemotherapy has been approved as adjuvant therapy for resectable NSCLC (49). Notably, oncolytic viral therapy can not only kill tumor cells, but also increase cytokines such as interferon gamma and interleukins, which are present in the TME, resulting in an improved innate immunologic response to tumor cells (123). The combination of oncolytic viral therapy and anti-PD-1/PD-L1 therapy might have the potential to enhance antitumor efficacy and reduce adverse events (123–125). Furthermore, angiogenic factors play important roles in inducing an immunosuppressive state and anti-angiogenic agents have been proven to enhance tumor immunity response. A clinical study has shown that the combined therapy of anti-angiogenic agents anlotinib and PD-1 inhibitor camrelizumab had promising efficacy and manageable toxicity in the treatment of NSCLC (126). Interestingly, the combination of different immunotherapy could achieve better therapeutic effect and outcomes. Chimeric antigen receptor T (CAR-T) cell therapy belongs to the immunotherapy that can specifically identify and kill tumor cells through the genetically modified T cells *in vitro* (127). More notable, the combination of CAR-T therapy and anti-PD-1/PD-L1 therapy has been proven to enhance antitumor efficacy and improve safety on cancer treatment (128, 129). Sanaz Taromi et al. reported that the combined therapy of CAR-T cells, anti-PD-1-antibody, and CD73 inhibitor can specifically eliminate chemo-resistant tumor stem cells and overcome SCLC-mediated T-cell inhibition in a humanized orthotopic SCLC mouse model (129).

Anti-PD-1/PD-L1 immunotherapy has been widely used in the treatment of lung cancer patients. However, a great proportion of patients could not exhibit sustained clinical response to the anti-PD-1/PD-L1 immunotherapy. It is of significance to screen out patients suitable for immunotherapy and to improve immunotherapy efficacy. Accumulating lines of evidence demonstrated that predictive biomarkers play an important role in predicting response and safety of anti-PD-1/PD-L1 therapy and in the identification of lung cancer patients who will benefit from anti-PD-1/PD-L1 immunotherapy. Current commonly used markers include PD-L1 expression level of tumor tissue, TMB, dMMR/MSI, and specific driver gene mutations. However, the predictive precision was affected by the intrinsic limitation of the markers when they are used alone to predict the response, and then the combination of multiple biomarkers is usually used in the prediction process (130–132). The surgical approach and a minimally invasive intervention to collect tumor tissues are commonly used traditional biopsy methods, while liquid biopsy can easily be retrieved from plasma or serum (102). Liquid biopsy has been developed to identify circulating cancer biomarkers including blood DNA-related biomarkers, blood cell-related biomarkers, and others such as PD-L1 and cytokines (133). Furthermore, novel techniques, such as high-dimensional single-cell analysis, have also been developed to predict response to anti-PD-1/PD-L1 immunotherapy (134).

Although anti-PD-1/PD-L1 therapy has achieved clinical success in the treatment of various cancer types, the immune-related adverse events (irAEs) should not be ignored (135). Among patients who received anti-PD-1/PD-L1 therapy, the incidence of any grade irAEs and severe grade irAEs was 26.82% (95% CI, 21.73–32.61; *I* ^2^, 92.80) and 6.10% (95% CI, 4.85–7.64; *I* ^2^, 52.00), respectively (136). The irAEs caused by immune checkpoint blockade thereby mainly involve abnormality on skin, gastrointestinal tract, endocrine glands, lung, liver, and other tissues. During nivolumab treatment, the most common irAE involves skin and gastrointestinal tract, with an incidence of approximately 13% (136). During pembrolizumab treatment, the most common irAE was hypothyroidism with an incidence of approximately 8% (136). Immune-related cholangitis was observed in patients receiving nivolumab and avelumab, which has raised concerns about liver damage induced by ICI drugs (137).

There remain challenges in the treatment of cancer using anti-PD-1/PD-L1 therapy, including screening out patients who may benefit from immunotherapy, improving therapeutic effect, and reducing the side/adverse effects. Therefore, more prospective studies are still warranted to validate the detailed function of biomarkers in clinical trials by increasing the tumor types and number of enrolled cancer patients. In the future, more efforts should be exerted to the screening of more predictive biomarkers that will help make an accurate therapeutic strategy and identify suitable patients who should be given the precision medicine to reduce their suffering from cancer. On the basis of anti-PD-1/PD-L1 therapy, the combined therapy also needs to be further optimized to improve the treatment of lung cancer with reduced adverse events.

## Author contributions

LW and ZY wrote the manuscript and drew the pictures with partial help from FG, YC, and JW. XZ and XD edited and revised the manuscript. All authors contributed to the article and approved the submitted version.
